# Through-time 3D radial GRAPPA for whole heart cardiac imaging

**DOI:** 10.1186/1532-429X-14-S1-P279

**Published:** 2012-02-01

**Authors:** Nicole Seiberlich, Katherine L Wright, Philipp Ehses, Mark Griswold

**Affiliations:** 1 Biomedical Engineering, Case Western Reserve University, Cleveland, OH, USA; 2 High-field Magnetic Resonance, Max Planck Institute for Biological Cybernetics, Tübingen, Germany; 3 Radiology, Case Western Reserve University, Cleveland, OH, USA

## Summary

Through-Time 3D Radial GRAPPA can be used to reconstruct 3D CINE images covering the whole heart in a single breathhold.

## Background

Through-Time Non-Cartesian GRAPPA has been previously demonstrated for real-time 2D cardiac imaging (Seiberlich, et al. Magn Reson Med. 2011 Feb;65(2):492-505.). This parallel imaging method works by acquiring several fully-sampled non-Cartesian datasets with a low temporal resolution, and using the coil sensitivity information from these datasets to reconstruct highly undersampled non-Cartesian data acquired in real-time. By modifying this through-time non-Cartesian GRAPPA method to reconstruct highly undersampled 3D data, whole heart 3D CINE images can be generated using data acquired in a single breathhold.

## Methods

A total of 20 fully-sampled 3D stack-of-stars radial datasets were acquired during free-breathing with no EKG gating using a 1.5T Siemens Espree and the following parameters: bSSFP sequence, TE=1.52ms, TR=3.04ms, matrix size = 128x128x20, projections/partition=128, FOV=300x300x90mm^3^, Flip Angle=45°, 5/8 Partial Fourier, 18 receiver channels. Segmented undersampled data (using only 16 projections/partition, an acceleration factor of R=8) were acquired with EKG gating and the above parameters during a breathhold for 15 heartbeats, resulting in 15 CINE frames. In order to perform the calibration, each of the time frames and partitions were employed as separate sources of calibration information; thus, a total of 300 repetitions could be used to generate the through-time GRAPPA weight sets. After reconstruction, the undersampled data yielded fully-sampled 3D CINE images, each with a temporal footprint of 48ms, an in-plane resolution of 2.3mm^2^, and a through-plane resolution of 6mm. The total acquisition time was 116s for the calibration and approximately 15 s for the breathhold CINE acquisition.

## Results

**Figure 1:**
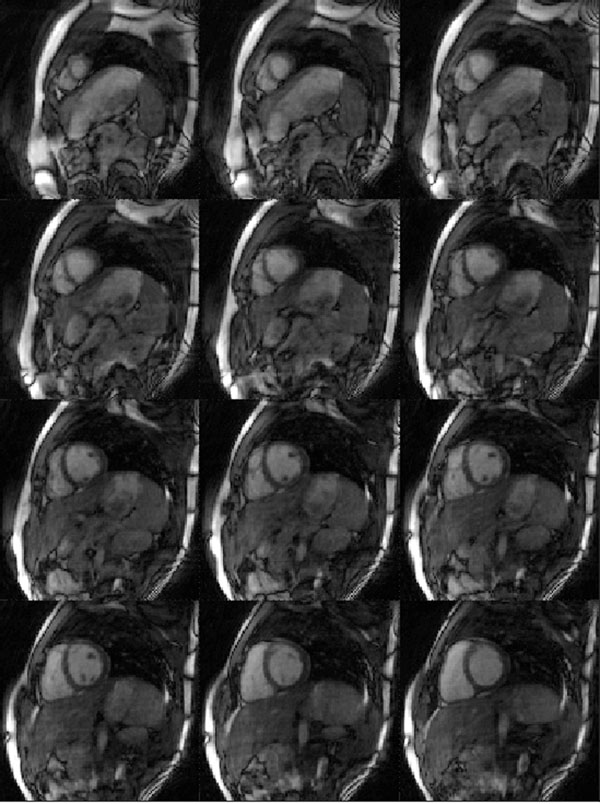
One of the 15 frames acquired in the 3D CINE dataset showing 12 partitions of the heart in diastole.

**Figure 2:**
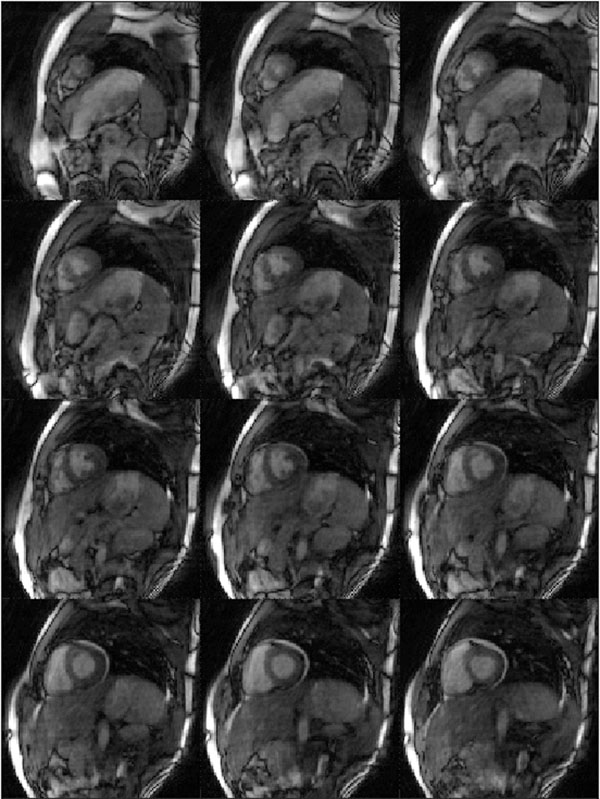
Another of the 15 frames acquired in the 3D CINE dataset showing 12 partitions of the heart in systole.

Example images from diastole and systole of one healthy volunteer are shown in Figures 1 and 2. It is important to note that these represent just two of the 15 CINE frames acquired in this dataset. Despite the high acceleration factor (R=8 in comparison to the fully-sampled calibration data), the images demonstrate only minor residual aliasing artifacts. Because a 3D dataset is acquired, the images from each partition can be shown in the same cardiac phase, which is challenging when using multiple breathholds to acquire several 2D CINE slices.

## Conclusions

By combining through-k-space calibration used in standard parallel imaging with through-time calibration, undersampled 3D radial CINE datasets can be reconstructed to yield CINE images at similar temporal resolutions as 2D CINE images with coverage of the entire heart in a single breathhold.

## Funding

The authors would like to acknowledge funding from Siemens Medical Solutions and NIH grants 1RO1HL094557 and 5K99EB011527.

